# Process and Outcome Evaluations of Smartphone Apps for Bipolar Disorder: Scoping Review

**DOI:** 10.2196/29114

**Published:** 2022-03-23

**Authors:** Iona Tatham, Ellisiv Clarke, Kelly Ann Grieve, Pulkit Kaushal, Jan Smeddinck, Evelyn Barron Millar, Aditya Narain Sharma

**Affiliations:** 1 Translational and Clinical Research Institute Faculty of Medical Sciences Newcastle University Newcastle upon Tyne United Kingdom; 2 National Specialist Adolescent Mood disorders Service Cumbria Northumberland Tyne and Wear NHS Foundation Trust Walkergate Park Newcastle upon Tyne United Kingdom; 3 Open Lab, Human Computer Interaction Urban Sciences Building Newcastle University Newcastle upon Tyne United Kingdom

**Keywords:** child and adolescent mental health, scoping review, bipolar disorder, mental health

## Abstract

**Background:**

Mental health apps (MHAs) provide opportunities for accessible, immediate, and innovative approaches to better understand and support the treatment of mental health disorders, especially those with a high burden, such as bipolar disorder (BD). Many MHAs have been developed, but few have had their effectiveness evaluated.

**Objective:**

This systematic scoping review explores current process and outcome measures of MHAs for BD with the aim to provide a comprehensive overview of current research. This will identify the best practice for evaluating MHAs for BD and inform future studies.

**Methods:**

A systematic literature search of the health science databases PsycINFO, MEDLINE, Embase, EBSCO, Scopus, and Web of Science was undertaken up to January 2021 (with no start date) to narratively assess how studies had evaluated MHAs for BD.

**Results:**

Of 4051 original search results, 12 articles were included. These 12 studies included 435 participants, and of these, 343 had BD type I or II. Moreover, 11 of the 12 studies provided the ages (mean 37 years) of the participants. One study did not report age data. The male to female ratio of the 343 participants was 137:206. The most widely employed validated outcome measure was the Young Mania Rating Scale, being used 8 times. The Hamilton Depression Rating Scale-17/Hamilton Depression Rating Scale was used thrice; the Altman Self-Rating Mania Scale, Quick Inventory of Depressive Symptomatology, and Functional Assessment Staging Test were used twice; and the Coping Inventory for Stressful Situations, EuroQoL 5-Dimension Health Questionnaire, Generalized Anxiety Disorder Scale-7, Inventory of Depressive Symptomatology, Mindfulness Attention Awareness Scale, Major Depression Index, Morisky-Green 8-item, Perceived Stress Scale, and World Health Organization Quality of Life-BREF were used once. Self-report measures were captured in 9 different studies, 6 of which used MONARCA. Mood and energy levels were the most commonly used self-report measures, being used 4 times each. Furthermore, 11 of the 12 studies discussed the various confounding factors and barriers to the use of MHAs for BD.

**Conclusions:**

Reported low adherence rates, usability challenges, and privacy concerns act as barriers to the use of MHAs for BD. Moreover, as MHA evaluation is itself developing, guidance for clinicians in how to aid patient choices in mobile health needs to develop. These obstacles could be ameliorated by incorporating co-production and co-design using participatory patient approaches during the development and evaluation stages of MHAs for BD. Further, including qualitative aspects in trials that examine patient experience of both mental ill health and the MHA itself could result in a more patient-friendly fit-for-purpose MHA for BD.

## Introduction

### Background

There are many critical factors that can influence the course and outcome of mental health disorders. Two key factors are (1) early and accurate identification of the first onset and subsequent relapses of the disorder, leading to the institution of appropriate management, and (2) access to appropriate treatment locations. For bipolar disorder (BD), the average delay between the onset of symptoms and the first institution of treatment can be as long as 10 to 15 years [1-3]. Between 35% and 50% of patients with mental health disorders receive no treatment because appropriate treatment locations are rare [4]. BD is no exception to this rule. A United Kingdom–based 2015 study found that the median diagnostic delay in the South London and Maudsley National Health Service (NHS) Trust was 62 days, with the median treatment delay being a further 31 days [5]. Research regarding pathways to redress these delays is urgently required, and with the potential to reliably scaffold processes and scale to both large numbers and remote locations, digital technology holds considerable potential to address these challenges.

In 2020, an estimated 6 billion smartphones were in use across the globe [6]. In the United Kingdom, there has been a 79% increase in the number of 5- to 15-year-old children owning mobile phones since 2015 [7]. Although smartphone ownership tends to be more common in high-income countries, as economies develop, the price of smartphones will decrease, and this correlation will reduce [6]. One form of digital technology that can capitalize on this increased smartphone usage globally is mental health apps (MHAs).

**Figure 1:**
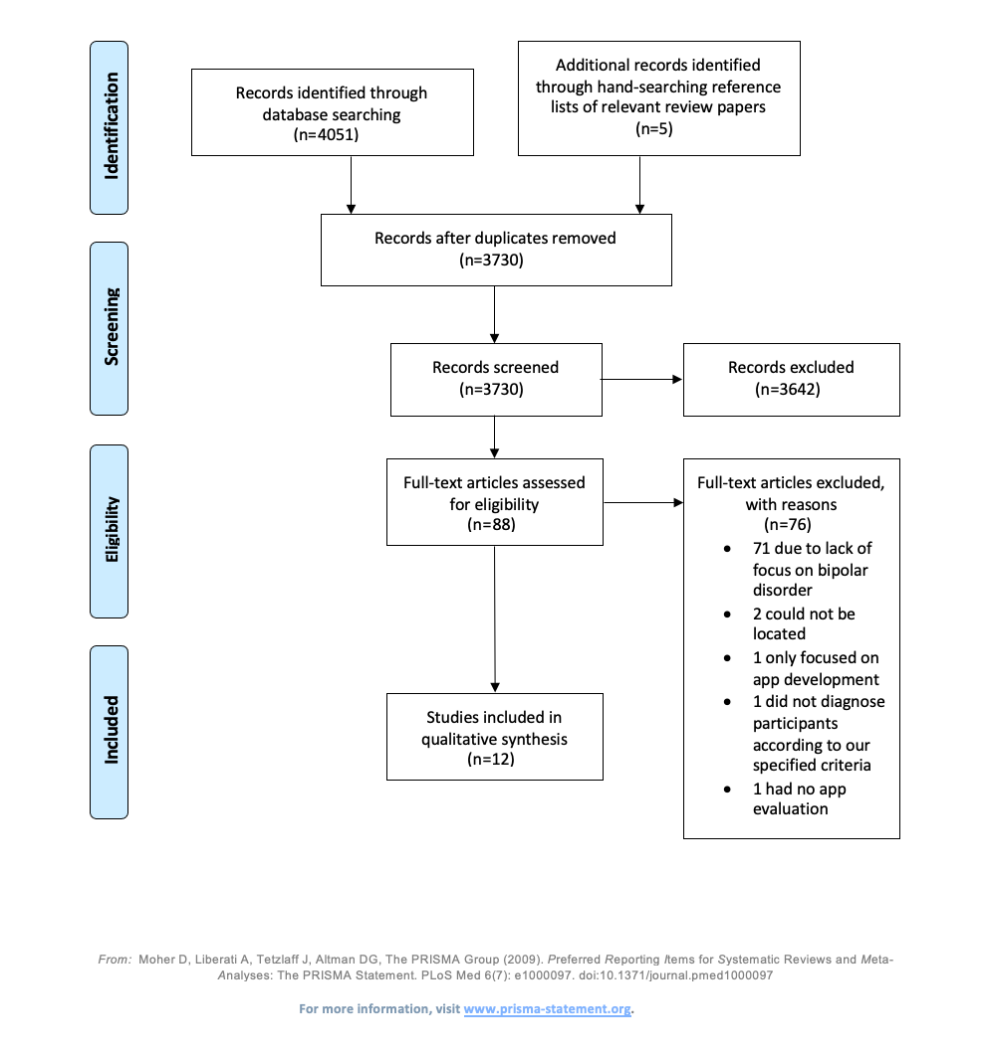
Preferred Reporting Items for Systematic Reviews and Meta-Analyses (PRISMA) flowchart for scoping reviews.

### Prior Work

Currently, MHAs can be seen to improve engagement and accessibility for individuals in rural areas where health care provision is increasingly difficult to access [8,9], and they are well accepted by service users [10]. So much so that financial incentives have been implemented for behavioral health information technologies in US policy [11]. Such advances in digital mental health care have now been adopted by clinicians in the treatment of common mental disorders in the United Kingdom [9]. Cost-effectiveness is crucial to health care, especially in a government-funded system as comprehensive as the NHS. Evidence suggests that the use of tele-psychiatry interventions reduced pressure on mental health services in low- and middle-income countries in comparison to a control group [12], but noted the importance of rigorous app evaluation. This is echoed by Tal et al [13], who described both the potential opportunities and risks posed by digital mental health, and how they must be balanced in order to achieve meaningful change.

The socioeconomic cost of BD in the United Kingdom is well recognized [14]. Previous literature suggests that the use of MHAs for BD can increase patient engagement and provide real-time symptom monitoring to allow for improved recognition of symptoms of relapse [15]. Subsequently, this reduces barriers to treatment, such as lack of resources and time. However, the efficacy of MHAs for BD is unclear [16], and a paucity of evidence in how to assess and evaluate MHAs for BD makes these statements difficult to qualify. To date, there is a lack of regulatory guidelines regarding MHAs, including those for BD, as health technologies are a relatively new resource within the NHS. This could potentially lead to unsafe use and practice [17]. Little is known about how MHAs (including those for BD) are developed and scrutinized, and studies predict that consumers, policymakers, health services, and funders will demand a robust evaluation process before funding, prescribing, or using these services [18].

As the development of an MHA for BD requires iterative processes with stakeholders outside of the academic and clinical research environment, process evaluation is important (in addition to more traditional outcome measure methods) to ensure the app remains user-friendly and functional without compromising clinical outcomes. This scoping review aims to address this research gap through mapping existing literature on process and outcome evaluation methods of MHAs for BD to increase the understanding around currently available evaluation tools and the latest practice.

### Aim

The aim of this scoping review is to systematically explore current process and outcome measures to identify the best practice for evaluating MHAs for BD. The focus is on apps for BD designed for individuals across the lifespan. Conducting a scoping review will allow health care systems to be more structurally informed on how to accurately evaluate the effectiveness of such technologies when implementing them into routine care [19]. The specific objectives of this scoping review, based on the detailed framework of Levac et al [20], were to map the available evidence and report on (1) process evaluation methods (ie, participant usability and functionality), (2) outcome measure methods (ie, data on how widely a measure is used and concordance of the target population with completing a measure), (3) outcome measures used to measure mental health improvement (eg, well-being measures), and (4) methods for best practice in the evaluation of MHAs for BD.

## Methods

### Overview

The review was informed by the Arksey and O’Malley 5-step framework [19], which was further developed by the Levac, Colquhoun, and O’Brien model [20]. This includes identification of a research question, study selection and criteria, data extraction, and content analysis. Employing this methodological framework will support in examining the broader field of the evaluation of MHAs for BD to identify the best practice.

### Search Strategy

A scoping search was initially performed in the following databases: PsycINFO, MEDLINE, Embase, EBSCO, Scopus, and Web of Science. Relevant search terms were identified from key papers, and the search strategy was developed iteratively in MEDLINE and then translated across the other databases, up to January 2021. Due to time constraints, grey literature sources were not investigated, and the search was limited to articles published in English, as no resources were available to undertake translation work. Broad search terms were used to reduce the likelihood of article omission. The complete search strategy for MEDLINE is available in Multimedia Appendix 1. The reference lists of included studies were hand searched for additional reports of relevance.

### Selection Criteria

For studies to be included in this review, they needed to meet the following inclusion and exclusion criteria. Studies were included if they (1) were related to BD, (2) targeted individuals across the lifespan, (3) included qualitative and/or quantitative evaluation methods and measures, (4) were published in the English language, (5) had no start date limit, (6) included any type of study design, (7) included participants with symptoms of BD or diagnosed with BD according to International Classification of Diseases-10, Diagnostic and Statistical Manual of Mental Disorders-IV, or Diagnostic and Statistical Manual of Mental Disorders-5, (8) included evaluation of the functionality of the MHA and/or evaluation of the participant outcomes, and (9) included any function (eg, screening, mood monitoring, or medication adherence). Studies were excluded if they (1) were based on a web-based intervention with no MHA counterpart, (2) included MHAs that were only psychotherapeutic intervention specific with no evaluation, (3) were based on MHA development only, and (4) included a participant population without symptoms of BD or not diagnosed with BD. Where systematic review papers were identified, these were not included. However, their reference lists were hand searched to identify primary articles relevant for inclusion.

Given that the aim of the study was to recognize the scope of research already conducted, both qualitative and quantitative research designs were included. As few studies focused on children and adolescents as their participant population, no age limits were applied.

### Selection Process

The search was completed by 2 researchers (IT and PK), who independently screened articles by the title and abstract against the inclusion criteria. Articles that fulfilled the inclusion criteria were then subjected to full-text screening by IT and PK. Conflicts were discussed with a third researcher (EBM) to reach consensus. Eight conflicts arose altogether, including 6 when screening the title, 1 when screening the title and abstract, and 1 when screening the full paper.

### Data Collection Process

Characterization of the data and the results were exported into a customized data extraction form that was piloted in a subset of included studies. Data extracted included study name, authors, year, country, study design, MHA for BD, whether the MHA for BD was independent or adjunctive, sample size, mean age of the participants, gender of the participants, inclusion/exclusion criteria for the participants, results, tools used, measures used, time points, and whether it addressed any of the 4 objectives.

### Data Synthesis and Quality Assessment

EC and IT analyzed the data using narrative synthesis and placed this in the context of the current literature to formulate conclusions. The studies were assessed using the Mixed Methods Appraisal Tool (MMAT) [21].

## Results

### Overview

The original database search yielded 4051 articles. Hand searching of relevant review articles was conducted, which yielded a further 5 articles. After duplicates were removed, 3730 articles remained. Screening of the title and abstract resulted in 3642 articles being excluded. The remaining 88 articles were then subjected to full-text screening, and 76 articles were excluded (71 due to a lack of focus on BD, 2 could not be located, 1 only focused on app development, 1 did not diagnose participants according to our specified criteria, and 1 had no app evaluation).

### Study Characteristics

Overall, 12 studies were identified as part of this review, which evaluated 7 MHAs for BD. Multimedia Appendix 2 describes the characteristics and assessment compliance of each study [22-33], and Multimedia Appendix 3 describes the results of the respective compliance with the standards set out in the MMAT [22-33]. Across all 12 studies, data from 435 participants were analyzed (343 with BD). Five out of the 12 studies stated the type of BD for 167 participants (112 had bipolar I disorder, 52 had bipolar II disorder, and 3 had bipolar disorder not otherwise specified). Eleven of the 12 studies provided the mean age (37 years) of the participants with BD. All 12 studies provided information on the gender (M:F of 137:206) of the participants with BD.

Assessment of the quality of all 12 studies (quantitative and mixed methods) was performed (IT and EBM) using the MMAT (version 2018) [21]. The results of their respective compliance with the standards set out in the MMAT can be found in Multimedia Appendix 3. Scores ranged from 20% to 100%. However, low-quality studies were not excluded in order to summarize the small pool of available literature.

### Process Evaluation

Six studies examined the self-perceived participant usability and functionality of the MHAs for BD. This examination ranged from detailed feedback questionnaires given to the participants [22,23] to participant feedback suggesting that a reminder prompt from the MHA for BD increased completion rates. Only 1 study mentioned functionality problems in MHAs for BD. Bardram et al [23] commented that MONARCA only worked 63 out of the 69 days of the study period and the information quality score was lower due to unresponsive error messages. The authors also noted that the Android market locked the app during the study period, negatively affecting the pattern of usage during that time.

Two studies recognized that technical problems were likely to arise and so implemented a system to solve these problems. Hidalgo-Mazzei et al [34] supplied participants with technical support via telephone, so they could contact the researcher for further assistance. A similar system was put in place by Schärer et al [24]. Subjects were able to report errors and receive immediate assistance by phone or personal communication. However, it was found that the MHA for BD required a certain amount of knowledge as a prerequisite, which restricted its use in comparison to a text message equivalent [24].

### Outcome Evaluation

A variety of validated outcome measures were used to evaluate the selected MHAs for BD. The most widely employed measure was the Young Mania Rating Scale (n=8) [35]. The Hamilton Depression Rating Scale [36] was applied 3 times, and the Altman Self-Rating Mania Scale [37], Quick Inventory of Depressive Symptomatology [38], and Functional Assessment Staging Test [39] were used on 2 occasions. The Coping Inventory for Stressful Situations [40], EuroQoL 5-Dimension Health Questionnaire [41], Generalized Anxiety Disorder Scale-7 [41], Inventory of Depressive Symptomatology [42], Mindfulness Attention Awareness Scale [43], Major Depression Index [44], Morisky-Green 8-item [45], Perceived Stress Scale [46], and World Health Organization Quality of Life-BREF [47] were all utilized just once. Other measures were assessed in 9 different studies, 6 of which used MONARCA. These measures included mood, sleep length, medication taken, activity level, irritability, mixed mood, cognitive problems, alcohol consumption, stress level, menstruation, individualized early warning sign, energy level, anxiety, elation, sadness, anger, speed of thoughts, and impulsivity. Mood and energy level were the most commonly utilized measures, being used 4 times each.

### Outcome Measure Methods

Eleven of the 12 studies presented a debate on the confounding factors affecting the efficacy of MHAs for BD. These confounding factors and the number of times they were mentioned in the 11 studies are shown in Table 1.

**Table 1:** Identified potential confounders for mental health app efficacy.

Potential confounder	Number of studies in which mentioned
Participants were mainly stable or euthymic	4
Participant insight when experiencing a manic phase varies	3
Sample size was too small	3
Length of study was too short	3
Low retention or adherence rate	2
Method of objective data collection was not robust enough	2
Patients were found to be capable of experiencing both manic and depressive symptoms concurrently	1
Questionnaires given were too simplistic	1
Opportunity of free-text input not given	1
Error in the app	1
Change in mobile phone communication habits	1
Low prevalence of manic symptoms	1
Potential sampling bias	1
Order of questions in the questionnaire did not vary and so was open to mindless input	1
Scales not delivered often enough	1
Participants switched the mental health app (MHA) off during the study	1
Participants may have chosen not to complete the surveys due to their mood	1
Subjective scales	1
The MHA gave daily confrontation with depressive symptoms	1
The MHA was not sensitive enough to manic or depressive symptoms	1
Participants were already involved in a medication adherence intervention	1

### Evaluation of MHAs for BD

Only 5 of the 12 studies commented on the future of the evaluation of MHAs for BD. Streicher et al [48] suggested that instead of measuring relapse or recurrence of affective episodes, a more sensitive measure would be assessment of mood instability or subsyndromal symptoms. They also commented that future research should include patients with bipolar disorder not otherwise specified, as this patient group may represent a large proportion of patients with BD. Hidalgo-Mazzei et al [34] acknowledged the low retention and adherence rates of MHAs for BD, and stated that researchers should focus on developing new approaches to motivate and engage patients with the intervention in the long term. The authors suggested adopting a user-centered design approach or incorporating gamification elements into a formal psychoeducation process.

Osmani et al [25] commented on the personalization of MHAs for BD, with a focus on physical activity; however, the authors found it difficult to generalize their results to the wider population. This was due to substantial variations between patients for both overall physical activity levels and physical activity levels within daily intervals. Therefore, the authors considered that an adaptive approach to user modeling would be better suited to detect early warning signs of the onset of episodes of BD and facilitate timely intervention. This involves personalizing goals and achievements around each patient’s individual needs. This has been evidenced in previous conference proceedings [48].

Schwartz et al [26] found that the generalizability of the results was limited due to a lack of a comparison group with a differing psychiatric diagnosis, which may exhibit overlapping symptoms. They recommended that future research should use additional comparison groups to better differentiate between symptoms.

Faurholt-Jepsen et al [27] proposed that emphasis should be placed on the differentiation between day-to-day difficulties and depressive symptoms. A positive reinforcing feedback mechanism may help minimize the negative processing bias and so, in theory, relieve the sustained depressive symptoms. They addressed the notion that it can be difficult for an intervention to have an effect on both depressive and manic symptoms, given the complexity of BD [27]. These suggestions are in keeping with the existing literature [49].

## Discussion

### Principal Findings and Comparison With Prior Work

The aim of this scoping review was to better understand how MHAs for BD are being evaluated, particularly in terms of the process of use and outcome measures. Due to the scarcity of studies evaluating MHAs for BD specifically, inferences for discussion have been assumed from studies evaluating general MHAs. This relies on the assumption that the functions are similar.

The need for effective and diligent evaluation of MHAs for BD is well established in the literature; no credentialing is currently required for their development and release. Karcher et al [50] warned that the “questionable content” and sparse evidence base of the myriad of current MHAs available warrant careful consideration. Effective robust evaluation systems would be required in aiding patients and practitioners in making individualized appropriate decisions regarding their role and treatment options in patient care. The NHS in the United Kingdom made considerable progress in this area when they launched their Digital Technology Assessment Criteria (DTAC) in February 2021. This provides a “simpler and faster assessment process to help give staff, patients, and the public confidence that the digital health tools they use meet NHS standards” [51]. The DTAC bring together legislation and good practice into a core document [52] that all digital health technologies have to meet in order to be recommended by clinical policy teams within NHS England and NHS Improvement. Though this goes so far to provide the public with centrally regulated technologies, clinicians may lack the knowledge and skills required for the effective recommendation of an MHA [25-27,43-54]. Mindfulness and meditation MHAs are the most commonly recommended by general practitioners in Australia [54]. However, the clinical presentation of BD and its specialist management may deter general practitioners from researching or recommending MHAs for its monitoring or management. Therefore, training health care professionals’ skills in identifying and selecting high-quality MHAs for BD would be beneficial for patients. If, as the literature suggests, the use of MHAs decreases service use [10], the financial benefit may outweigh the cost of the additional training required.

One barrier in the development of MHA evaluation systems is the adherence rate. O’Connell [55] reported that 74% of users stop engaging with an MHA after only 10 uses. Low adherence reduces the confidence with which researchers can generalize their results [56]. Aforementioned personalization and gamification of MHAs for BD have been recommended; however, engagement can tail off once the initial novelty effect of the feature has subsided. It has been suggested that a change in the communication approach may help to solve this problem. Kenny et al [57] surmised that it is possible that in studies where participants (specifically young people) are aware of the importance of their input to achieve the research objective, engagement levels may be higher. This brings into focus the importance of participatory co-design and co-production of MHAs for BD. Eight of our 12 studies mentioned adherence rates (adherence rates were not applicable in 2 studies, and another 2 studies failed to mention the rates), with the average rate being 84%. In fact, Tsana et al [28] experienced 91% adherence over the first 3 months of the study period and 81.9% in the following 9 months. The variability between the studies by O’Connell [55] and Tsanas et al [28] illustrates that there is still work to be done to achieve successful and reliable compliance with such apps.

Interestingly, one reason for the low utilization of MHAs for BD may be decreased motivation, which is often a key feature of depressive episodes [57]. Previous literature suggests that “communities of practice” around an MHA can improve long-term concordance, with social interaction and communal use (whether in person or digitally), encouraging users to continue to access it [49,50]. Integrating a “days since last updated” screening tool would help identify early relapse in the usage of MHAs for BD, and aid in assessing clinical usability [18].

Torous et al [58] interviewed adolescent patients to identify which factors would be useful to bear in mind for MHA development. The results included safety, engagement, functionality, social interaction, awareness, gender, and participative engagement by young people. One study strongly suggested the abandonment of randomized controlled trials as a method of evaluating and improving apps, and instead called for iterative participatory research or single case designs [59]. As such, MHA developers would work in collaboration with patients throughout the design and development process in order to gain regular ecologically valid feedback so that relevant appealing prototypes are established. This could take the form of consumer-used tools or accreditation portals [60]. Then, when pilot and nonpilot studies are performed, both qualitative and quantitative data could be obtained in order to receive valuable feedback in how to further improve the app. The role of randomized controlled trials can then be established in validating the MHAs at later stages. Torous et al [58] theorized further reasons for low engagement, including poor usability, lack of a user-centric design, privacy concerns, and lack of trust. Another study found that MHA efficiency, effectiveness, memorability, and learnability and cognitive load were major usability barriers to continuing MHA use [61]. This evidence lends further strength to the argument that streamlining of the usability of MHAs should be at the core of future iterative development stages of MHAs in order to increase adherence rates and improve the ecological validity and reliability of evaluations.

Torous et al [58] also recognized accessibility as a factor to consider when developing MHAs. The Office for National Statistics stated that in 2018, 10% of the UK adult population were internet “nonusers” [62], meaning they had never used the internet or had not used it in the last 3 months. This brings the idea of the digital divide into the spotlight and shows the complexities it brings with it along with merely accessibility. The digital divide cannot be solved by just providing patients with devices, as they will also need digital literacy skills to use the device to its full potential. Even though Torous et al [58] interviewed adolescents, their study does illustrate the need to consider the skill level of the intended audience. Ennis et al [63] found that lack of technological skills was the reason for nonengagement with computers and mobile devices. Furthermore, Ennis et al found that only a quarter of their 121 participants reported familiarity and easy access to smartphones. Therefore, throughout the MHA evaluation process, patients’ skill sets, in addition to access, should be taken into consideration.

As well as employing participative engagement in MHA development, improving user awareness can come from creative measures, such as describing or advertising the MHA appropriately. Over a quarter of MHAs for depression failed to mention depression in the title or description [64].

Moving to the user-identified priority of safety [29,58], third parties obtaining confidential information is considered the greatest threat to MHA use [65]. Tools are available that can increase device security [50]; however, threats to privacy are continually emerging and endangering data security. For example, identity cannot be confirmed unless a video-calling app is used and personal devices are easily lost or stolen, leaving data vulnerable [50]. Karcher et al [50] determined that the greatest threat to patient privacy in MHA use was the possibility of confidential information being shared with third parties, whether via patient or clinician devices. Hacking of secure devices and new viruses were also identified as challenges to a secure patient database on MHAs. As well as being its own point for consideration when overcoming the challenges of evaluating the clinical effectiveness of MHAs, the complex legal and ethical considerations involved in MHA use are a consideration for clinicians themselves [50]. This has been highlighted in recent news, where contact tracing apps used to help curb the spread of COVID-19 have been the subject of widespread debate.

From a more pragmatic standpoint, MHA cost may be a factor in choosing the right MHA for BD, with 76% of people surveyed reporting interest in using their mobile phones for mental health monitoring and self-management if the MHA was free of charge [15]. Moreover, Larsen et al [66] reported that an MHA clinically relevant for depression is being removed from the market every 2.9 days. This furthers the challenge faced by both patients and clinicians in trying to identify a relevant and appropriate MHA for BD. If the MHA for BD is to be paid for and could be removed from the market without warning, it is difficult to justify its recommendation and purchase.

### Limitations

This review had its own limitations. Only 7 MHAs for BD were evaluated, somewhat limiting the generalizability of the results of this review. Furthermore, only 5 studies commented on the future of the development and evaluation of MHAs for BD.

### Conclusion

The studies in our review focused on patient monitoring as an indicator for process and outcome evaluation in MHAs for BD. They based their conclusions on whether the app improved assessment scores rather than interviewing patients on their experience of using the app. Although this is suggested to be a reliable way of measuring the process and outcome values, as modern medicine shifts to holistic patient-centered care, more emphasis should be put on users’ experiences rather than quantitative outcomes. In the long term, this will make patients feel respected and involved in the design of MHAs for BD, increasing adherence rates in both the short and long term.

Personalized medicine is a rapidly emerging movement in the field of health care. It is defined as a move away from the “one size fits all” approach to treatment, with new approaches and targeted therapies allowing for flexibility in the management of diseases. With this in mind, more MHAs for BD should be easily available in order to encourage patient choice and freedom to choose an MHA that is best suited to them. At the moment, MONARCA dominates the market, reducing the range and scope of MHAs for BD. As NHS England suggests [60], it can be difficult for an intervention to address both depressive and manic symptoms given the complexity of BD. This is all the more reason to develop a wider variety of apps, with some apps perhaps only focusing on either mania or depression.

The field of MHAs for BD shows promise in both improving patient care and creating a more cost-effective health care service [10]. However, as with any new development in health care, it must be appropriately evaluated and regulated. By encouraging patient co-design and co-evaluation, we can develop a new frontier in personalized digital health, while improving patient experience and care.

## Appendix

Multimedia Appendix 1 MEDLINE and PsycINFO searches.

Multimedia Appendix 2 Study characteristics.

Multimedia Appendix 3 Study designs and outcomes.

## Abbreviations

BD: bipolar disorder
DTAC: Digital Technology Assessment Criteria
MHA: mental health app
MMAT: Mixed Methods Appraisal Tool
NHS: National Health Service
