# Supplementary material for: Process and Outcome Evaluations of Smartphone Apps for Bipolar Disorder: Scoping Review
Source: J Med Internet Res. 2022 Mar 23;24(3):e29114. doi: 10.2196/29114 (PMC8987951; doi:10.2196/29114)
Supplement: Multimedia Appendix 1 [file jmir_v24i3e29114_app1.docx]

## **Multimedia Appendix 1.** MEDLINE and PsycINFO searches.

| **Search Number** | **Search Terms** | **Operator** |
| --- | --- | --- |
| 1 | Mental Health/ |  |
| 2 | Exp mental disorders/ |  |
| 3 | (Psychological adj4 (illness* or health or disorder* or problem*)).mp. |  |
| 4 | 1, 2, 3 | OR |
| 5 | Mobile Applications/ |  |
| 6 | (Mobile adj4 app*).mp. |  |
| 7 | 5, 6 | OR |
| 8 | 4, 7 | AND |
