# Supplementary material for: Process and Outcome Evaluations of Smartphone Apps for Bipolar Disorder: Scoping Review
Source: J Med Internet Res. 2022 Mar 23;24(3):e29114. doi: 10.2196/29114 (PMC8987951; doi:10.2196/29114)
Supplement: Multimedia Appendix 2 [file jmir_v24i3e29114_app2.docx]

## **Multimedia Appendix 2.** Study characteristics.

| **Name of study** | **Author** | **Year** | **Country** | **Study Design** |
| --- | --- | --- | --- | --- |
| Mood instability in bipolar disorder type I versus type II- continuous daily electronic self-monitoring of illness activity using smartphones [29] | Faurholt-Jepsen, Ritz, Frost, Mikkelsen, Christensen, Bardram, Vinberg, Kessing | Published 2015 | Denmark | Randomised |
| Designing Mobile Health Technology for Bipolar Disorder: A Field Trial of the MONARCA System [23] | Bardram and Faurholt-Jepsen | MONARCA arm of study ran May- August 2011 | Denmark | Field Trial |
| Smartphone data as objective measures of bipolar disorder symptoms [30] | Faurholt-Jepsen, Frost, Vinberg, Christensen, Baradram, Kessing | Published 2014 | Denmark | Pilot |
| Smartphone application for the analysis of prosodic features in running speech with a focus on bipolar disorders: system performance evaluation and case study [31] | Guidi, Salvi, Ottaviano, Gentili, Bertschy, Rossi, Scilingo, Vanello | Published 2015 | France | System performance evaluation |
| Psychoeducation in bipolar disorder with a SIMPLe smartphone application: feasibility, acceptability and satisfaction [22] | Hidalgo-Mazzei, Mateu, Reinares, Murru, del Mar Bonnin, varo, Valenti, Undurraga, Strejilevich, Sanchez-Moreno, Vieta, Colom | Published 2016. Conducted March to August 2015 | Spain | Feasibility, acceptability and satisfaction |
| Monitoring activity of patients with bipolar disorder using smartphones [25] | Osmani, Maxhuni, Grunerbl, Lukowicz, Haring, Mayora | Published 2013 | Denmark | Uncontrolled, not randomised, monocentric, prolective, observational study |
| Daily longitudinal self-monitoring of mood variability in bipolar disorder and borderline personality disorder [28] | Tsanas, Saunders, Bilderbeck, Palmius, Osipov, Clifford, Goodwin, De Vos | Published 2016 | UK | Observational |
| Daily mood monitoring of symptoms using smartphone in bipolar disorder: A pilot study assessing the feasibility of ecological momentary assessment [26] | Schwartz, Shultz, Reider, Saunders | Published 2016 | USA | Two-arm, parallel group, observational study |
| Daily electronic self-monitoring in bipolar disorder using smartphones - the MONARCA I trial: a randomized, placebo-controlled, single-blind, parallel group trial [27] | Faurholt-Jepsen, Frost, Ritz, Christensen, Jacoby, Mikkelsen, Knorr, Bardram, Vinberg, Kessing | Published 2015 | Denmark | Randomised, placebo-controlled, single-blinded, parallel group design |
| Validation of life-charts documented with the personal life-chart app - a self-monitoring tool for bipolar disorder [24] | Schärer, Krienke, Graf, Meltzer, Langosch | Published 2015 | Germany | Prospective, multi-site natural observation study |
| Smartphone data as an electronic biomarker of illness activity in bipolar disorder [32] | Faurholt-Jepsen, Vinberg, Frost, Christensen, Bardram, Kessing | Published 2015 | Denmark | Randomised control trial |
| Using smartphones to monitor bipolar disorders symptoms: a pilot study [33] | Beiwinkel, Kindermann, Maier, Kerl, Moock, Barbian, Rossler | Published in 2016 | Germany | Pilot, adjunctive |
