# Supplementary material for: Process and Outcome Evaluations of Smartphone Apps for Bipolar Disorder: Scoping Review
Source: J Med Internet Res. 2022 Mar 23;24(3):e29114. doi: 10.2196/29114 (PMC8987951; doi:10.2196/29114)
Supplement: Multimedia Appendix 3 [file jmir_v24i3e29114_app3.docx]

## **Multimedia Appendix 3.** Study designs and outcomes.

| Name of study | MHA-BD evaluated | MMAT study design | % compliance |
| --- | --- | --- | --- |
| Mood instability in bipolar disorder type I versus type II- continuous daily electronic self-monitoring of illness activity using smartphones [29] | MONARCA | 2 | 60 |
| Designing Mobile Health Technology for Bipolar Disorder: A Field Trial of the MONARCA System [23] | MONARCA | 5 | 40 |
| Smartphone data as objective measures of bipolar disorder symptoms [30] | MONARCA | 4 | 60 |
| Smartphone application for the analysis of prosodic features in running speech with a focus on bipolar disorders: system performance evaluation and case study [31] | Psyche | 4 | 40 |
| Psychoeducation in bipolar disorder with a SIMPLe smartphone application: feasibility, acceptability and satisfaction [22] | SIMPLe | 4 | 100 |
| Monitoring activity of patients with bipolar disorder using smartphones [25] | MONARCA | 4 | 80+ |
| Daily longitudinal self-monitoring of mood variability in bipolar disorder and borderline personality disorder [28] | Mood Zoom [63] | 4 | 60 |
| Daily mood monitoring of symptoms using smartphone in bipolar disorder: A pilot study assessing the feasibility of ecological momentary assessment [26] | DREAM | 5 | 20 |
| Daily electronic self-monitoring in bipolar disorder using smartphones - the MONARCA I trial: a randomized, placebo-controlled, single-blind, parallel group trial [27] | MONARCA | 2 | 100 |
| Validation of life-charts documented with the personal life-chart app - a self-monitoring tool for bipolar disorder [24] | PLC | 4 | 100 |
| Smartphone data as an electronic biomarker of illness activity in bipolar disorder [32] | MONARCA | 4 | 100 |
| Using smartphones to monitor bipolar disorders symptoms: a pilot study [33] | SIMBA | 4 | 80+ |
